# Traumatic Penile Injury: From Circumcision Injury to Penile Amputation

**DOI:** 10.1155/2014/375285

**Published:** 2014-08-28

**Authors:** Jae Heon Kim, Jae Young Park, Yun Seob Song

**Affiliations:** ^1^Department of Urology, Soonchunyang University Hospital, College of Medicine, Soonchunhyang University, Seoul, Republic of Korea; ^2^Department of Urology, Korea University Ansan Hospital, Korea University College of Medicine, Ansan, Republic of Korea

## Abstract

The treatment of external genitalia trauma is diverse according to the nature of trauma and injured anatomic site. The classification of trauma is important to establish a strategy of treatment; however, to date there has been less effort to make a classification for trauma of external genitalia. The classification of external trauma in male could be established by the nature of injury mechanism or anatomic site: accidental versus self-mutilation injury and penis versus penis plus scrotum or perineum. Accidental injury covers large portion of external genitalia trauma because of high prevalence and severity of this disease. The aim of this study is to summarize the mechanism and treatment of the traumatic injury of penis. This study is the first review describing the issue.

## 1. Introduction

Among the hospitalized patients, the admission rate of genitourinary trauma patients has been assumed to be 2–10% and one-third or two-thirds of them were found to have the injury on external genitalia [1]. Male is prone to have external genitalia more frequently than female because the male is more exposed to violence or extreme exercise [1].

External genitalia injury can be categorized as accidental in origin including during circumcision and as other traumatic origins including animal bite, gunshots, or self-mutilation [2]. Most injuries of the male genitalia include penetrating injury with foreign bodies owing to psychiatric illness and abnormal sexual behaviors [3].

In this study, we reviewed the traumatic injury of external genitalia in male by describing diverse traumatic nature of the injury. This issue has never been reviewed before.

## 2. Etiologies and Classifications

To date, there is no standard classification for external genitalia injury. Main reasons for this include the diverse natures of injury mechanism and various anatomical landmarks. Rashid et al. reported the classification of male genitalia injury by anatomical location [4]. Type I injury includes distal portion of the penis with proximal part of the penis being preserved. Type II injury includes severe injury on shaft of penis with penile crus being preserved. Type III injury includes the injury when urethral catheterization is necessary with external urethral part being preserved. Type IV injury includes the injury when suprapubic cystostomy is needed [4]. However, this classification could not reflect the nature of injury mechanism such as penetrating or strangulation injury. The other classification for male external genitalia could be suggested as adult or pediatric injury, iatrogenic. In this report, authors suggest the classification by pediatric and adult injury and also including self-mutilation injury. For detailed etiologies, there are circumcision injury, animal bite injury, strangulation injury, penetration injury, zipper injury, and penile fracture and self-mutilation injury.

### 2.1. Pediatric Injury

Pediatric injury of penis includes circumcision injury, animal bite injury, and zipper injury. Reports of penile injury in the pediatric population are sporadic and often related with sexual abuse. Most reports about the pediatric penile injury were based on a small number of cases [5–11].

The types and severity of nonsexual pediatric penile injury vary from a small injury to total emasculation. Owing to their rarity and disparity there is no universal therapeutic strategy in their management.

The etiologies for pediatric penile injury are different from that of adult penile injury [6–11]. El-Bahnasawy and El-Sherbiny reported the pediatric penile injury in large population group of 64 patients [5]. The most common cause was circumcision (63%), followed by hair-tie strangulation. The most common sequel after replantation was the loss of the coronal sulcus, in which buccal graft has been used with the successful outcome [12].

#### 2.1.1. Circumcision Injury

Circumcision is one of the most common operations in urology, which is usually a safe and simple procedure with low morbidity. However, serious complications can occur because unprofessional practice performs it [13]. The penile injury from circumcision is diverse: from infections to disfigurement or partial to total amputation of the penis.

Gee et al. reported the postoperative complication rate as 0.2–0.6%, which ranges from bleeding, lymphedema, fistula formation, and iatrogenic hypospadias to the partial or complete amputation of the glans penis [14, 15]. El-Bahnasawy and El-Sherbiny [5] reported the largest series of pediatric penile injury. Sixty-four boys with penile injury were hospitalized over 20 years and among them 43 boys (67%) had penile injury caused by circumcision.

Although circumcision is regarded as a minor surgical procedure, it is not free of complications. Urologists have to pay more attention to reducing the complication by circumcision. Penile injury by circumcision also can have lifetime functional, psychological, and cosmetic sequel.

#### 2.1.2. Animal Bite Injury

The sequel of penile injury by animal bite is related with initial severity of the wound. Pediatrics has more tendencies to be exposed to animal bite injury, of which the most common cause is a dog bite [16]. Although most of the injuries are not severe condition, total or nearly total amputation of penis is being reported [17]. Nowadays, infective complications occur less because most wounds are initially treated properly with antibiotics [18]. Initial treatment strategy includes sufficient clean irrigation, excision of infective wound, and administration of broad spectrum antibiotics [19]. In some cases, vaccination against tetanus and rabies is needed [1, 18, 19].

#### 2.1.3. Zipper Injury

Penile zipper injury occurs most commonly in boys with phimosis; in particular it occurs when the redundant foreskin gets entrapped during hastened dressing or undressing. Entrapment of the foreskin within the zipper itself is the most problematic condition [20]. Most of the cases are detected in an earlier stage of trauma but in rare cases, delayed presentation and comorbidities may worsen the treatment outcome.

Penile zipper injury is a challenging management disease, especially when the injured penis is complicated by medical comorbidities. Delayed disease includes the edema and infection of entrapped skin, which complicates the treatment [9].

Various methods of zipper removal have been described including both surgical and nonsurgical methods. Documented methods are manual disengagement of the zipper with lubrication [21], cutting the median bar with bone cutter or hacksaw [20, 22], dismantling the fastener [23–25], or removal of the entrapped skin [26, 27].

### 2.2. Adult Injury

Most of adult penile injuries are penile fracture and other causes include strangulation injury and penetrating injury.

#### 2.2.1. Penile Fracture

Penile fracture is a rupture of the tunica albuginea, which is the outer membrane of the penile corpora cavernosum, occurring during penile erection. The etiology of this injury can be divided into two parts: sexual and nonsexual causes. The primary mechanism of this injury is an abrupt, blunt trauma by forceful bending of the erect penis over the pubic bone or perineum [28]. For sexual causes, vigorous intercourse and masturbation were reported [28–30] and, for nonsexual causes, falling off from bed, placing an erect penis in the underwear, and spontaneous fracture during urination were reported [28–31]. Most penile fractures are underreported because of culture issues.

Penile fracture could be diagnosed based on clinical presentation and physical examination. It rarely needs radiologic evaluation except in cases with gross hematuria that requires retrograde urethrography [32, 33]. Physical examination reveals swelling of the penile shaft with eggplant deformity, discoloration, and deviation of the penile shaft. In cases when the hematoma is contained within Buck's fascia, the rolling sign which is a palpable clot felt direct over the tear in the tunica albuginea could be manifested [30]. Surgical exploration warranted when penile fracture is suspected because either clinically or radiologically penile fracture could not be excluded [34]. If the depth of injury extended into Buck's fascia, bloody discharge can extravagate into the subcutaneous plane of the scrotum, perineum, or pubic areas, resulting in significant swelling with discoloration. Concomitant urethral injuries have been reported to be 3% to 38 [35]. Total urethral rupture also could happen and its rate is up to 2.32%, which needs end to end urethral anastomosis [29].

The location of the fractured site is usually transverse and unilateral in nature [36]. There could be many complications such as erectile dysfunction and urethral stricture, which depends on the time interval since initial injury [36]. Many reports support the immediate surgical repair offers, which yield better long-term results than conservative treatment [33–36]. The current standard treatment for penile fracture is immediate surgical repair, because of low rate of subsequent morbidity. Immediate surgical repair results in an excellent outcome in sexual behavior among 90% of patients [37].

During surgical repair of penile rupture, urethral catheterization could facilitate anatomical orientation, which makes easier discrimination from a large hematoma [37].

#### 2.2.2. Strangulation Injury

Penile strangulation is not common and only a few reports have been published up to now. Most common cause for penile strangulation is foreign body object which compress circumferentially by metallic or nonmetallic material. Nonmetallic and thin objects are easy to remove. The causing objects for penile strangulation documented are usually heavy metal rings, hammer-head, and plastic bottle neck, sprockets, or plumbing cuff [38]. Metal objects are relatively difficult to remove and to cut the metal objects is the most common method documented [38, 39]. However, in real practice, most medical facilities are not equipped with appropriate cutting machine. Furthermore, cutting the metallic object is a time-consuming process [38]. Cutting tools described are an iron saw, orthopedic equipment, and a high-speed diamond-tipped dental drill [40, 41].

Other methods to solve the penile strangulation include aspiration method and delving method [42]. Another useful method is using a string with a glandular puncture, which is easier and quicker than the previous methods [40, 41]. However, in cases of combining with the foreskin edema, decompression with puncture both on foreskin and glandular lesion should be performed [40, 41].

#### 2.2.3. Penetrating Injury

Among the penetrated injured sites by foreign body, urethra is the most common involved site besides areas of the penis including penile skin, glans, and corpus cavernosum [43].

The main reason for penetrating injury into penis is self-insertion of foreign body on purpose of sexual eroticism [44]. Most cases of penetrating injury in penis can be diagnosed through physical examination and retrograde urethrography; computed tomography or ultrasound test is seldom necessary [45]. Various foreign bodies, such as a screw, a wire, and a safety pin, have reported in the urethra [46]. The most appropriate method for removing the penetrated international body depends on the size and depth of penetration of the material.

## 3. Psychiatric Impact

Total penile amputation is an uncommon penile injury [47, 48]. However, about 87% of the patients reported had psychiatric problems. Self-amputation of the penis is known as Klingsor syndrome [47, 49]. The extent of self-mutilation varies in its severity from supericial injury to total amputation or total emasculation [50, 51]. Klingsor syndrome is a disease of self-mutilation by a psychiatric patient, often suffering from religious delusions [50–52].

These psychiatric patients have paranoid schizophrenia along with command hallucinations [51, 53]. This disease is a urological emergency, which requires urgent surgical corrections because the associated hemorrhage can be torrential and life threatening.

Genital self-mutilation injury has a common connotation with eating behavior disorders such as anorexia and bulimia. Self-mutilation is a way of expressing and dealing with deep distress, anger, dissociation, and emotional pain to have self-purification [54]. However, self-purification by self-mutilation does not last very long [54].

Large et al. [55] suggest that one of the primary causes for major self-mutilation is the individual's first psychotic break. In cases with schizophrenia, the degree of injury extent can be rather bizarre and potentially very harmful. Patients with schizophrenia are known to attempt self-mutilation due to command hallucination, catatonic excitement, or associated depression [24].

Genital injury by self-mutilation involves injury to the penis, the scrotum, and the testicles. The type of injury varies from simple skin laceration to total amputation of the penis and testis.

## 4. Treatment

The first case with macroscopic penile replantation was reported in 1929 by Ehrlich [47]. Cohen et al. reported the first microvascular replantation of penis in 1977. Approximately more than 70% of cases were treated with macroscopic replantation since 1970.

The distal penile stump has no circulation because the arterial supply consists of the branches of pudenda artery, dorsal artery, deep artery, bulbourethral artery, and accessory pudenda artery. Variation is present in the origin, distribution, and symmetry of these arteries.

Replantation of a penile stump without reestablishing the arterial blood site could be regarded as a graft. Hence it should survive by imbibition, obtaining nutrients from the adjacent graft by diffusion [56]. Graft is successful method because the dorsal and urethral arteries represent an excellent source of vascularity to the glans and corpus spongiosum [57].

Without vascular reestablishing the arterial blood supply, the circulation ater macroscopic repair could be reestablished through the spongy tissue of the penis as a graft [58].

This microsurgical replantation of the penis depends on corporal sinusoidal blood flow, which could act as diffusion for the composite graft. However, by this process complications of skin necrosis, fistula formation, loss of sensations, and erectile dysfunction have been reported [47].

The current concept of treatment choice is microvascular replantation for penile amputation because it yields better cosmetic restoration, physiological micturition, preservation of sensation, and erectile function.

The development of microsurgical techniques has improved the rate of successful clinical outcome regarding the penile replantation [59]. This method also has some weak point that it is not always possible to identify deep dorsal arteries, veins, or nerves in pediatric patients [56]. Furthermore, this procedure requires special equipment, instruments, and training, which are not always available in generalized hospital.

Belinky et al. invented a method to use the distal urethra to cover the distal unroofed corpus cavernosum, but this process requires a relatively healthy urethra and long penile stump to obtain satisfactory sexual and cosmetic result [60]. Mazza et al. developed a two-stage technique using a scrotal fasciocutaneous flap, which is tubularized and sutured to the distal end of the penis. This process provides good cosmetic results but has some shortcomings like higher expense due to two-stage operation and a high rate of metal stenosis [61]. Buckle mucosa could be used to reconstruct the distal parts of the cavernosum but this method has a tendency to have contractures [62].

Pediatric phalloplasty has some controversies including the indication about the age, size, and, especially, neophallic growth during puberty [1]. Performing penis reconstruction in childhood is crucial to minimize the emotional impact by this surgery because normal like appearance is important for children especially during puberty to prevent emotional stress and to achieve favorable genital identity [63].

## 5. Complications

Superficial or partial penile injury can be treated with suturing and wound dressing after exploration. More extensive injuries including urethral and corpus cavernous can be treated by free transfer flaps and different grafts. Penile amputation, whether it is partial or total, requires complex and skilled reconstructive techniques including phalloplasty [12, 64, 65].

Expeditious and prudent postoperative care is needed to avoid delayed complications such as infection, curvature, erectile dysfunction, unrecognized urethral injury, and chronic pain. Severe penile injury might be associated with adjacent comorbidity involving the scrotum, pelvis, buttocks, and thighs. In these scenarios, delicate surgical skill with staged treatment is needed [5].

The aim of the reconstruction in penile injury is to embody an esthetically acceptable shape, to obtain normal or near normal functional outcomes including erection and sensation, and to minimize the postoperative sequel including fistulae or urethral strictures.

## 6. Comment

Owing to the specific location and mobility of penis, severe injury on penis is rare. The severity of penile injury could be judged by the depth of the penis: glans or penile skin, corpus cavernosum, and urethra. However, in cases of inatrogenic injury, severe injuries could be often observed. Furthermore, although the penis has mobility and protected by direct trauma due to its particular area, it is more prone to injury in erection state [66, 67].

During erection state, tunica albuginea becomes thinner, and it is highly susceptible to penile injury. The mean arterial pressure of corpus cavernosum during erection is 100 mmHg. To overcome the tensile strength to be ruptured, mean arterial pressure is needed to be over 1500 mmHg [68].

To reduce the possibility of the sequel such as devastating deformities such as deviation or shortened penis and functional impairment, these patients have to be treated by expert surgeons as soon as possible severe penile is injury can be defined when patients have two or more injury of the following components: penile skin, glans, corpora cavernous, and urethra.

The location of the amputated site is very critical landmark for treatment strategy in managing penile amputation. When the amputation occurs at the shaft of the penis, microvascular replantation is recommended. Most amputations are less documented because those injuries are repaired immediately. The glans amputation could be successfully reattached if it is managed within eight hours [69]. However, if the injury happens to be detected even after eight hours, the stump could be connected successfully resulting in favorable cosmetic and functional results [69, 70]. There have been many techniques for glans reconstruction after complete or partial amputation.

Recently, Faydaci et al. reported the successful outcome of treatment in penis amputation by circumcision [71]. They performed hyperbaric oxygen therapy after primary anastomosis. Considering that oxygen plays an important role for wound healing, hyperbaric oxygen therapy can increase the angiogenesis and stimulate the proliferation of fibroblast [72].

## 7. Conclusion

To date, there are no specific guidelines for the treatment of severe penile injury because the injury mechanism is a complex and multifaceted subject. In this review, authors have described the various penile injuries, which have relatively higher incidence. Physicians have to keep in mind that the goal of treatment of penile injury is to achieve normal-like appearance, reduce functional damage such as erectile dysfunction and sensory loss, and minimize the postoperative sequel. Furthermore, pediatric penile injury has to be approached with delicate and prudent care plan.
